# A Comparison Between Dexamethasone and Clonidine as Adjuvants to Levobupivacaine in the Supraclavicular Approach to the Brachial Plexus Block: A Double-Blind Study

**DOI:** 10.7759/cureus.46776

**Published:** 2023-10-10

**Authors:** Sapna Yadav, Kirtika Yadav, Jaishri Bogra, Monica Kohli, Rajni Gupta

**Affiliations:** 1 Anesthesiology, Sanjay Gandhi Postgraduate Institute of Medical Sciences (SGPGIMS), Lucknow, IND; 2 Pain Management, Era Medical University, Lucknow, IND; 3 Anesthesiology, King George's Medical University (KGMU), Lucknow, IND; 4 Anesthesiology and Critical Care, King George's Medical University (KGMU), Lucknow, IND

**Keywords:** brachial plexus block, adjuvant, dexamethasone, analgesia, clonidine

## Abstract

Objective: The objective of this clinical study is to compare the efficacy of adding dexamethasone or clonidine as an adjuvant drug to levobupivacaine in supraclavicular brachial plexus block (BPB) with regard to the onset and duration of sensory and motor blocks along with duration of postoperative analgesia.

Background: Brachial plexus block (BPB), with or without general anesthesia, has been used widely for multiple upper limb surgical procedures, by virtue of its efficacy in terms of cost-effectiveness, efficiency, safety margins, and good postoperative analgesia. Various adjuvant drugs have been described to potentiate the analgesic effect of local anesthetic agents such as epinephrine, clonidine, dexamethasone, dexmedetomidine, or midazolam.

Materials and methods: This is a prospective, randomized, double-blind study in which a total of 90 American Society of Anesthesiology (ASA) physical status I and II patients of either sex, aged between 18 and 60 years, were scheduled for elective upper limb surgical procedures under supraclavicular BPB. They were divided into three equivalent randomized groups with 30 patients in each group. The patients were administered either normal saline 2 mL (in group L) or clonidine 0.5 mcg/kg body weight (in group LC) or dexamethasone 8 mg (in group LD) with 30 mL of 0.5% levobupivacaine. The time of onset and duration of sensory and motor blockades along with the time duration of analgesia were compared.

Results: All groups were equivalent as per demographic data. The time duration for onset of sensory and motor blocks was comparable among all three included groups (12.77±2.60 minutes and 20.80±3.25 minutes, 15.93±2.08 minutes and 22.43±3.07 minutes, and 12.57±2.62 minutes and 22.47±3.10 minutes for group L, LC, and LD, respectively). The time duration of analgesia and motor blockade was significantly prolonged in the dexamethasone group (1195.33±50.01 minutes and 1173.17±43.57 minutes) and moderately prolonged in the clonidine group (696.33±36.74 minutes and 674.67±34.33 minutes) when compared to levobupivacaine group (416.33±35.98 minutes and 397.00±35.12 minutes), and the difference was statistically significant (p<0.001).

Conclusion: Dexamethasone appears to be a superior adjuvant drug to clonidine for brachial plexus block via supraclavicular approach as it provides prolonged duration of motor block with lesser requirement of postoperative analgesia and lack of adverse effects.

## Introduction

Brachial plexus block (BPB) renders optimum operating conditions for both elective and emergency upper limb surgical procedures (orthopedic, plastic, and reconstructive) by imparting muscle relaxation, sympathetic block, intraoperative hemodynamic stability, and augmented postoperative analgesia with minimal side effects. It also enhances better preservation of cognitive functions in the geriatric population, decreases the chances of aspiration by maintaining intraoperative laryngeal and pharyngeal reflexes, and decreases postoperative complications by avoiding intubation. Additionally, it also offers better postoperative analgesia without unwarranted sedation and opioid use, thereby facilitating early mobilization and discharge. The effect of BPB using a single local anesthesia injection may begin to diminish after a few hours. Many adjuvants [1] such as epinephrine, clonidine, dexamethasone, dexmedetomidine, or midazolam [2,3] are used in combination with local anesthesia to provide rapid onset and increase the duration of BPB.

This study was done to compare the efficacy of dexamethasone and clonidine as an adjuvant to 0.5% levobupivacaine in supraclavicular BPB.

## Materials and methods

After approval from the Institutional Ethical Committee (reference code 90th ECM II B-Thesis/P22), informed consent was taken from all the included patients after discussion of the study, procedure, and expected outcome in their own language. This prospective, randomized, double-blind study was conducted on 90 patients of either sex, aged between 18 and 60 years, with American Society of Anesthesiology (ASA) physical status I and II, posted for upper limb surgeries. The exclusion criteria comprised hypersensitivity to the local anesthetic agent, infection at the local site, coagulopathy, severe systemic disorders, pregnant and lactating patients, and those who had contraindications to either steroid or clonidine.

Patients were divided into three groups based on the use of a combination of anesthetic agents, viz., group L (30 mL 0.5% levobupivacaine + 2 mL normal saline), group LC (30 mL 0.5% levobupivacaine + clonidine (0.5 mcg/kg body weight) + normal saline to make it 32 mL), and group LD (30 mL 0.5% levobupivacaine + 8 mg (2 mL) dexamethasone). After a detailed pre-anesthetic checkup, patients were taken to the operating room, and an 18 G intravenous cannula was inserted into the non-operating hand. The patients were connected to the multichannel monitor for monitoring of vital parameters including heart rate, non-invasive blood pressure, respiratory rate, and oxygen saturation. All patients were pre-medicated with 4 mg IV ondansetron.

Patients were placed in a supine position with their heads turned away from the site of the block. Under aseptic precautions, skin infiltration was done with an injection lignocaine 2% at the site of the block before block placement. Under proper aseptic conditions, all included patients were given supraclavicular BPB using a peripheral nerve stimulator (0.8 mA). On the localization of the brachial plexus, aspiration for blood was performed before incremental injections of a total volume of 32 mL of solution. Block was evaluated using six parameters: onset of sensory block, onset of motor block, duration of analgesia, duration of sensory block, duration of motor block, and any complications.

The onset of sensory block was defined as the time from injection to complete loss of sensation in major peripheral nerve distributions (radial, ulnar, medial, and musculocutaneous nerves). Sensory nerve block was evaluated by pinprick test using the blunt end of a 27 G needle at 0, 2, 5, 10, 15, 20, and 30 minutes. Sensory block was classified according to the following three-point scale: 0 = no block (normal sensation), 1 = partial block (decreased sensation), and 2 = complete block (no sensation). The palmar surface of the little and index fingers were used to evaluate for a sensory block along the median and ulnar nerve in the hand, respectively. To evaluate the radial nerve block, the dorsal thumb surface was assessed. The onset of motor block was defined as the time from the completion of injection to complete paralysis. Motor block was evaluated by finger abduction and adduction (ulnar nerve), extension of the wrist, thumbs up (radial nerve), flexion of the arm at the elbow, supination of the forearm (musculocutaneous nerve), and "OK" sign (median nerve). Motor block was evaluated at 0, 10, 20, and 30 minutes and ranked according to the Lovett rating scale [4]. The duration of analgesia is defined as the time interval from the onset of the block to the time when the patient begins to experience pain. It was considered that the analgesic action of the drugs was terminated and rescue analgesia (intravenous 100 mg tramadol) was given. The duration of analgesia was monitored as per visual analog score (VAS) (ranging from 1-10) for pain at half-hourly intervals for the first 10 hours and then hourly up to 24 hours. Evaluation of analgesia as per VAS was already explained to all participants. The duration of motor block is calculated as the time interval between complete paralysis and complete recovery of muscle power (return of motor power to Lovett rating scale = 6). The duration of sensory block is defined as the time interval between the onset of complete sensory block and return of normal sensation (sensory score = 0). Patients were also supervised for any medication-related side effects and any procedure-related complications.

Randomization of the patients was done by computer-generated block randomization method for allocation among the three groups. Evaluation for the aforementioned parameters was conducted by the principal investigator who was blinded to the drugs used for the brachial plexus block. Drugs were prepared by a designated trial coordinator, and the block was given by the principal investigator.

The sample size was calculated with the help of the institutional statistics team. The statistical analysis for the study was done using the Statistical Product and Service Solutions (SPSS) software version 16.0 (IBM SPSS Statistics, Armonk, NY). The data was reported as mean with standard deviation (SD) and numbers with percentages. The means of the continuous variables (age, weight, and length of surgery) were collated in three groups using analysis of variance (ANOVA), whereas the demographic data for the categorical variables (sex) was compiled using the chi-square test. A p-value of <0.05 was considered to be significant.

## Results

All three groups were equivalent (Table 1) with respect to demographic data and gender distribution in our study. There is no significant (p>0.05) difference in age, gender, and weight among the groups.

**Table 1:** Basic characteristics of the patients a: unpaired t-test, b: chi-square test, SD: standard deviation

	Group L (n=30)	Group LC (n=30)	Group LD (n=30)	p-value
Age in years, mean±SD	31.70±9.41	32.93±9.53	29.17±8.93	0.28^a^
Gender				
Male, number (%)	23 (76.7)	21 (70)	19 (63.3)	0.53^b^
Female, number (%)	7 (23.3)	9 (30)	11 (36.7)	
Weight in kg, mean±SD	59.33±6.44	59.27±6.68	57.17±5.79	0.32^a^

**Figure 1:**
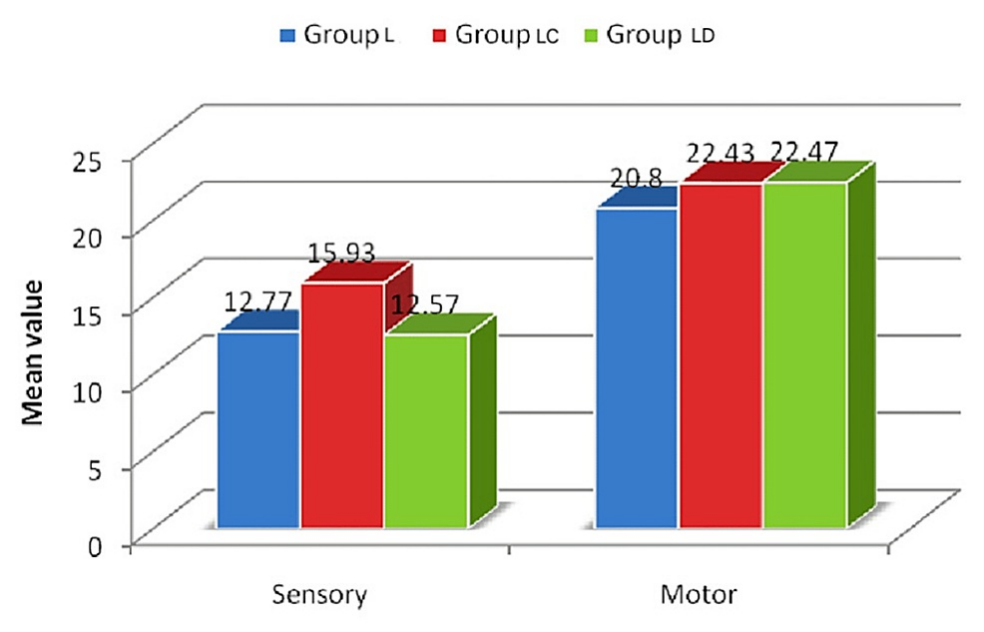
Comparison of time of onset among the groups x-axis: types of block, y-axis: time in minutes

The onset time (Figure 1, Table 2) of sensory and motor blocks in group LD (12.57±2.62 minutes and 22.47±3.10 minutes) was similar to group LC (15.93±2.08 minutes and 22.43±3.07 minutes) and group L (12.77±2.60 minutes and 20.80±3.25 minutes) with no significant difference (p>0.05).

**Table 2:** Comparison of time of onset among the groups 1: ANOVA test, ANOVA: analysis of variance

Time of onset in minutes	Group L (n=30)	Group LC (n=30)	Group LD (n=30)	p-value^1^
Sensory	12.77±2.60	15.93±2.08	12.57±2.62	0.06
Motor	20.80±3.25	22.43±3.07	22.47±3.10	0.07

**Figure 2:**
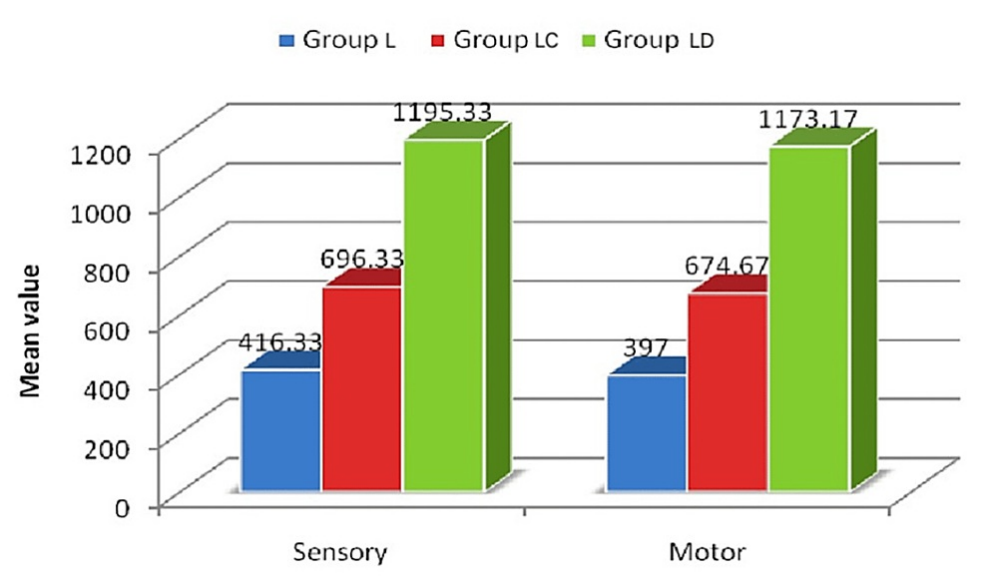
Comparison of duration of blockade time among the groups x-axis: types of block, y-axis: time in minutes

In our study, the duration (Figure 2, Table 3) of sensory block and motor block was significantly prolonged in group LD (1195.33±50.01 minutes and 1173.17±43.57 minutes) versus group LC (696.33±36.74 minutes and 674.67±34.33 minutes) and group L (416.33±35.98 and 397.00±35.12 minutes) with statistically significant p-value (p<0.0001).

**Table 3:** Comparison of duration of blockage time among the groups 1: ANOVA test, ANOVA: analysis of variance, *: significant, a,b: p=0.0001 (post hoc comparison tests)

Duration in minutes	Group L (n=30)	Group LC (n=30)	Group LD (n=30)	p-value^1^
Sensory	416.33±35.98^a^	696.33±36.74^a^	1195.33±50.01^a^	0.0001*
Motor	397.00±35.12^b^	674.67±34.33^b^	1173.17±43.57^b^	0.0001*

**Figure 3:**
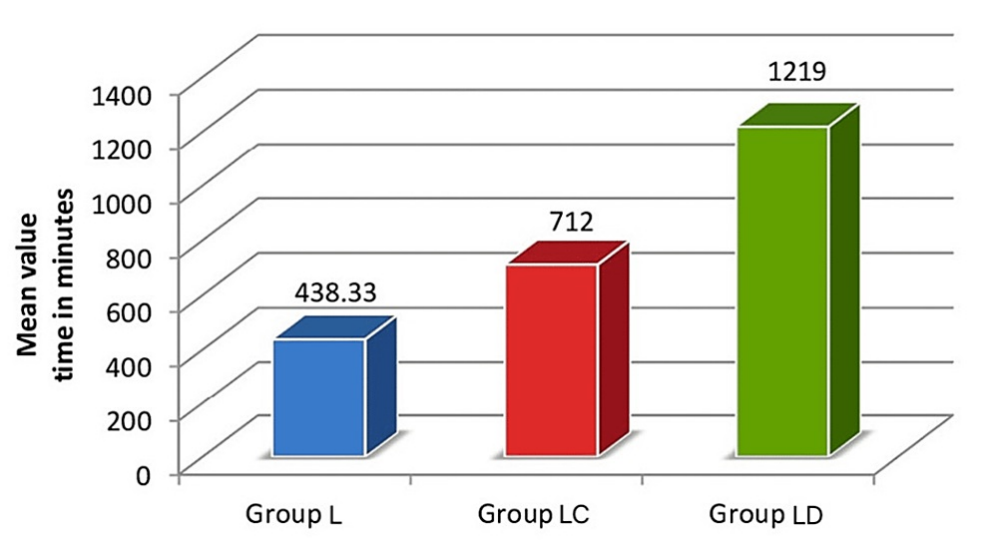
Comparison of time of rescue analgesia among the groups x-axis: included groups, y-axis: time in minutes

Figure 3 and Table 4 show the comparison of the time of rescue analgesia among the three groups. The analysis of variance revealed that the time of rescue analgesia is significantly (p=0.0001) different among the groups. The post hoc multiple comparison tests showed that the time of rescue analgesia is significantly different in each group, being higher in group LD when compared with group LC and group L.

**Table 4:** Comparison of time of rescue analgesia among the groups 1: ANOVA test, ANOVA: analysis of variance, *: significant, a: p=0.0001 (post hoc comparison tests), SD: standard deviation

Group	Time in minutes (mean±SD)
Group L	438.33±36.82^a^
Group LC	712.00±43.48^a^
Group LD	1219.00±49.69^a^
p-value^1^	0.0001*

Complications

Three patients who were administered clonidine in group LC developed bradycardia (heart rate less than 50 beats per minute) and were given 0.5 mg atropine IV. No other complications were noted in any of the patients.

## Discussion

In the present study, the supraclavicular approach was chosen for BPB in all three groups. It is associated with rapid onset, provides consistent and reliable anesthesia with analgesia, and is proven to be a safe technique. We used dexamethasone and clonidine as adjuvants to levobupivacaine for supraclavicular BPB. The onset of time of sensory and motor blocks was similar among all three arms of the study. Although both adjuvants prolonged the duration of analgesia as compared to levobupivacaine alone, the duration of analgesia with dexamethasone was significantly longer. The time of rescue analgesia was also significantly higher in the dexamethasone group.

Dewees et al. [5] compared interscalene block to supraclavicular BPB block using paresthesia and observed a higher incidence of complete sensory and motor blocks with supraclavicular block and a lower incidence of complications. Lanz et al. [6] showed that supraclavicular BPB results in a more homogenous block compared to interscalene block, which causes a preferential block of cephalad portions, and axillary block, which blocks caudal portions.

The time of onset for sensory and motor blocks in group LD was similar to group LC and group L (p>0.05) in our study. Similar results were observed in several earlier published studies [7-9]. Dar et al. [7] published a study that showed a significantly early onset of sensory and motor blocks in the dexamethasone group compared to the ropivacaine group (p<0.05). They also documented that the addition of 8 mg (preservative-free) dexamethasone to 0.5% ropivacaine in supraclavicular BPB has markedly prolonged the duration of pain relief in the dexamethasone group (14.5±0.3 hours) as compared to ropivacaine alone group (8.3±0.4 hours) (p<0.001).

Corticosteroids have been shown to prolong the nerve block effect by impeding the transmission of pain-carrying type-c myelinated fibers and repressing the ectopic neuronal discharge [10]. Corticosteroids are also known to modify the function of potassium channels of excitable cells.

Our findings also equate well with other latest published studies, such as those by Tandoc et al. [11], who documented significant accentuation of analgesic effect in interscalene BPB by addition of dexamethasone to bupivacaine. However, a direct comparison is difficult because of the use of different anesthetic drugs and the different blocks being studied.

Tandoc et al. [11] in 2011 documented equal efficacy in extending analgesic effect and motor blockade, whether 4 mg or 8 mg of dexamethasone is added to 0.5% bupivacaine (40 mL) in BPB via interscalene approach. We chose the dose of dexamethasone as 8 mg, consistent with the doses reported in the literature.

Our results for group LD are comparable with a recent meta-analysis done by Choi et al. [12] in 2014, which included nine trials. Out of 801 patients in these trials, 393 received dexamethasone doses ranging from 4 to 10 mg. They concluded that perineural administration of dexamethasone with local anesthetic prolongs analgesia and motor blockade effects in brachial plexus block with no observed adverse events.

When performing supraclavicular BPB, Parrington et al. [13] in 2010 demonstrated that adding 8 mg dexamethasone to mepivacaine increased the median duration of the supraclavicular block from 228 minutes to 332 minutes. Another study done in 2010 by Vieira et al. [14] found that adding 8 mg dexamethasone to a mixture of bupivacaine with 1:200,000 epinephrine and clonidine 75 mcg increased interscalene block duration from 833 minutes to 1457 minutes (1.7-fold prolongation). Our study also confirmed that the addition of 8 mg dexamethasone increased the block duration from 416.33±35.98 minutes in group L to 1195.33±50.01 minutes in group LD with a p-value of <0.0001.

Clonidine is a highly lipid-soluble molecule that easily crosses the blood-brain barrier to interact with alpha-2 adrenergic receptors at both spinal and supraspinal levels within the central nervous system, producing its analgesic effect. Clonidine possibly augments the sodium channel blockade action of local anesthetics by opening up the potassium channels, leading to membrane hyperpolarization, a state in which the cell becomes unresponsive to any excitatory input [15].

Many studies have validated that perineural administration of clonidine is superior to subcutaneous or intramuscular (im) injections [15], implying that the local anesthetic augmenting effect of clonidine is effectuated at the level of neuron [16,17]. This fact also explains the discrepancy seen in different types of nerve blocks, presumably related to the rate and extent with which the injected anesthetic agents penetrate the nerve.

In the present study, we have used 0.5 mcg/kg clonidine as an adjuvant. Singelyn et al. [18] reported that the minimum dose of clonidine added to mepivacaine to prolong the duration of anesthesia and analgesia after axillary BPB without any serious adverse effects is 0.5 mcg/kg, and they found no added advantage by exceeding the dose of clonidine to 1.5 mcg/kg.

Our results for group LC are comparable with a recent meta-analysis done by Popping et al. [3], who observed that clonidine, when added to intermediate or long-acting local anesthetics for single-shot peripheral nerve or plexus blocks, prolongs the duration of analgesia and motor block by about two hours. In the present study, the addition of clonidine prolonged the duration of analgesia from 416.33±35.98 minutes in group L to 696.33±36.74 minutes in group LC without any adverse effects.

Our results for group LC are also comparable with various other studies done by Rohan et al. [19], Ali et al. [20], Kohli et al. [21], and Kulkarni et al. [22], who also documented a significant prolongation of sensory and motor blockade without any alteration in hemodynamic stability with the addition of clonidine to local anesthetics used in BPB.

In our study, the baseline sedation score was 2 according to the Ramsay sedation scale when patients were taken in the operation theater (OT) (before any anesthetic intervention), as all patients were calm and cooperative, and the sedation score remained the same throughout the procedure; similar findings were seen in earlier studies [18,19]. However, several previous studies [19-22] had shown a high level of sedation with clonidine, which might be due to higher doses of clonidine used in their study.

Our observations are in accordance with a similar study published in 2015 by Kishore et al. [8,23], who compared the addition of dexamethasone in a dose of 8 mg (group D), clonidine in a dose of 1 mcg/kg (group C), and saline 2 mL (group S) with 15 mL of 0.5% bupivacaine in BPB by supraclavicular approach and observed that dexamethasone turned out to be a better adjuvant as compared to clonidine, as it significantly extend the analgesia duration, without any significant side effects.

Dexamethasone has clearly outperformed clonidine as a potent adjuvant (in terms of onset and duration of analgesia and anesthesia) when used in supraclavicular brachial plexus block as documented by latest studies done by Naveen Kumar et al. [24], Swetha et al. [25], Aggarwal et al. [26], Agarwal et al. [27] and Muni et al. [28].

In the present study, we found that the duration of analgesia was considerably prolonged in group LD (1195.33±50.01 minutes) and group LC (696.33±36.74 minutes) when compared to group L (416.33±35.98 minutes). Although the use of both clonidine and dexamethasone has shown a prolongation of analgesic effect when compared with group L alone, the duration of analgesia with dexamethasone was significantly higher (almost three times compared to group L). This was statistically more significant (p<0.0001), and this feature resulted in decreased postoperative use of analgesics in group LD.

An important shortcoming of our study lies in the fact that we measured the dose of clonidine based on the body weight, whereas the dose of dexamethasone was not calculated with respect to body weight and was fixed at 8 mg as the majority of the studies were done with this dose. Additionally, the equipotent dose of dexamethasone and clonidine could not be calculated. Also, an extended follow-up of the patients was not done, and thus, long-term complications could not be studied.

## Conclusions

Recent studies have highlighted the importance of the use of dexamethasone in brachial plexus blocks. However, the use of dexamethasone and clonidine as adjuvants is still justified in the present era while working in peripheral medical colleges of second-/third-tier cities in developing countries where the supply of dexmedetomidine is limited. Also, considering the side effects of dexmedetomidine (such as hypotension and bradycardia), the use of dexamethasone is still preferred when working in operation theaters (OT) with limited resources. Ultrasound is also not easily available in peripheral medical colleges in OT, so peripheral nerve stimulators are still functional in peripheral centers.

To conclude, dexamethasone proves to be a better adjuvant as compared to clonidine with levobupivacaine in brachial plexus block via the supraclavicular approach, given its availability, cost-effectiveness, longer duration of analgesia, lack of adverse effects, lesser requirement of postoperative analgesia, and more patient satisfaction. In terms of the onset of sensory and motor blocks and maintaining hemodynamics, dexamethasone and clonidine are equally effective.
